# Chemical fingerprinting of Korean ginseng (*Panax ginseng*) and American ginseng (*Panax quinquefolius*) using multi-platform metabolomics and taste profiling

**DOI:** 10.1007/s10068-026-02137-5

**Published:** 2026-04-08

**Authors:** Namhee Lee, Jung-Woo Lee, Yejin Kim, So-Jeong Kim, Kyong-Hwan Bang, Unyong Kim, Sang Beom Han, Jeehye Sung, Joon Hyuk Suh

**Affiliations:** 1 Department of Food Science and Technology, College of Agricultural and Environmental Sciences, University of Georgia, 100 Cedar Street, Athens, GA 30602 USA; 2 Department of Herbal Crop Research, Rural Development Administration, National Institution of Horticultural and Herbal Science, Eumseong, 27709 Republic of Korea; 3 Department of Food Science and Biotechnology, Gyeongkuk National University, 1375 Gyeongdong-Ro, Andong-Si, Gyeongsangbuk-Do 36729 Republic of Korea; 4 Department of Pharmaceutical Analysis, College of Pharmacy, Chung-Ang University, 84 Heukseok-Ro, Dongjak-Gu, Seoul, 06974 Republic of Korea

**Keywords:** Korean ginseng (*Panax ginseng*), American ginseng (*Panax quinquefolius*), Metabolomics, Electronic tongue, Quality control, Authentication

## Abstract

Diverse ginseng species exhibit highly similar morphological features, leading to potential concerns of adulteration. Difference in chemical composition, combined with sensory qualities, could be key to distinguishing between ginseng species and enabling their quality control. However, comprehensive metabolomic comparisons linking chemical profiles to sensory traits remain limited. This study aimed to differentiate Korean ginseng (*Panax ginseng*) and American ginseng (*Panax quinquefolius*) using multi-platform metabolomics approach combined with multivariate statistics by analyzing different tissue parts (whole, body, and root). Korean ginseng was overall enriched in sucrose, citric acid, acetic acid, *β*-panasinsene, and ginsenoside Ra2, whereas American ginseng contained higher succinic acid, *β*-farnesene, quinquenoside R1. Electronic tongue profiling further distinguished taste attributes, with Korean ginseng showing stronger sweetness and bitterness and American ginseng exhibiting higher sourness. These integrated findings identified discriminant markers and demonstrated the potential of integrated metabolomics and sensory analysis for ginseng authentication and quality assessment.

**Supplementary Information:**

The online version contains supplementary material available at 10.1007/s10068-026-02137-5.

## Introduction

Ginseng (*Panax* spp.) is one of the most valued medicinal plants worldwide, traditionally consumed for its health-promoting and adaptogenic properties. Among more than ten recognized species in the *Panax* genus, Korean ginseng (*Panax ginseng*) and American ginseng (*Panax quinquefolius*) are the most commercially important, serving as key raw materials for medicine, dietary supplements, and functional foods. The global market for ginseng was valued at USD 873 million in 2024 and is projected to reach USD 1.33 billion by 2032 (Research and Markets, 2025). Given this growing global demand and high economic value, ensuring product authenticity and species integrity has become increasingly important for maintaining consumer confidence and regulatory compliance.

Because of their high market value, similar appearance and close phylogenetic relationship, adulteration and substitution of American ginseng for Korean ginseng have often occurred in commercial products (Komatsu et al., 2001). Subsequent market surveys revealed that nearly half of products labeled as Korean ginseng were replaced by American ginseng (Wallace et al., 2012). A global review further demonstrated that one in four commercial ginseng products was adulterated through species substitution or replacement of roots with other plant parts, emphasizing the need for robust biochemical authentication to ensure quality and consumer safety (Ichim and De Boer, 2021). Reliable analytical methods are therefore essential to distinguish *Panax* species to ensure product integrity and consumer confidence in the expanding ginseng market.

Previous studies comparing *Panax* species have employed various metabolomics platforms to characterize their chemical diversity. Liquid chromatography–mass spectrometry (LC–MS) and Proton nuclear magnetic resonance (^1^H-NMR) based analyses have revealed distinct metabolic profiles between Korean and American ginseng, enabling reliable species discrimination (Lee et al., 2009; Leung et al., 2007; Li et al., 2010; Nguyen et al., 2016; Yang et al., 2016; Yuk et al., 2016). Comparative profiling has also uncovered tissue- and cultivation- dependent variation in ginsenoside composition (Chen et al., 2020, 2017). Across these studies, species differentiation has been primarily attributed to variation in characteristic ginsenosides. Recent untargeted LC–MS analysis further identified discriminant metabolites associated with species and environmental variation (Ji et al., 2023), highlighting the need for studies that control environmental factors to better define intrinsic species-specific metabolite traits. Volatile profiling using gas chromatography–mass spectrometry (GC–MS) has likewise demonstrated clear species separation (Cho et al., 2012; Kim et al., 2022), with sesquiterpenes serving as major discriminant compounds between Korean and American ginsengs. In addition, electronic-tongue (e-tongue) studies have demonstrated rapid and effective discrimination of *Panax* species and detection of adulteration or geographical origins through sensor-based taste profiling (Cui et al., 2015; Dong et al., 2017; Tian et al., 2021). Despite these advances, most previous studies have relied on a single analytical platform, and integration of multiple metabolomic datasets remains limited. Furthermore, controlling environmental factors is important for defining intrinsic species-specific metabolite traits (Ji et al., 2023).

To comprehensively understand differences in ginseng species, this study employed a multi-platform metabolomics approach to characterize metabolic variations between Korean and American ginseng cultivated in the same field under identical environmental conditions in South Korea. Primary metabolites such as sugars and organic acids were profiled to evaluate taste-related metabolic variation, while volatile compounds were analyzed to capture species-specific aroma characteristics. Untargeted LC–MS metabolomics was further conducted to examine the overall discriminant primary and secondary metabolites. In addition, e-tongue analysis was applied to support the interpretation of metabolomic data in terms of flavor. Through this multi-platform strategy, the present study aimed to establish metabolomic framework for species authentication and to elucidate the biochemical basis underlying interspecific variation. The findings provide new insights into the integrated metabolic signatures that define the unique characteristics of Korean and American ginseng, contributing to improved quality evaluation and authenticity assurance in the ginseng industry. By combining multiple analytical platforms with sensory profiling, this study advances beyond single-platform approaches and offers a more comprehensive basis for ginseng species authentication.

## Materials and methods

### Plant materials and sample preparation

The Korean ginseng and American ginseng materials used in this study were germplasm accessions collected and maintained by the National Institute of Horticultural and Herbal Science (NIHHS), Rural Development Administration (RDA) (Eumseong, Chungbuk Province, South Korea). Species identity was verified by NIHHS personnel based on taxonomic/morphological characteristics during germplasm maintenance, and each accession was assigned a unique NIHHS/RDA identification code (IT number). The *P. quinquefolius* accessions corresponded to IT numbers 357369–357,371, and the *P. ginseng* accessions corresponded to IT numbers 239610–239612. Seeds of each species were sown in November 2015 in an experimental field (36°94′17″ N, 127°74′87″ E), where seedlings were cultivated for one year. In 2016, one-year-old seedlings were transplanted into the same field and subsequently grown under identical agronomic conditions for five years. Within each species, plants representing the above accessions were harvested and pooled (mixed) prior to analysis. Ten plants per species were randomly selected, washed with tap water, flash-frozen in liquid nitrogen, and stored at –80 ℃ until analysis. Samples were analyzed as whole roots, root bodies, or fine roots depending on the experimental design.

### Experimental design

Multiple analytical platforms were applied to characterize metabolites in ginseng (i) free sugars, (ii) organic acids, (iii) volatile compounds, (iv) untargeted metabolomics, and (v). e-tongue. All analytical details, including chemicals, reagents, metabolite standards, and specific procedures, are provided in the Supplementary Material.

### Statistics

One-way analysis of variance (ANOVA) was conducted using R software (https://www.r-project.org). Principal component analysis (PCA), partial least squares discriminant analysis (PLS-DA) and orthogonal (O) PLS-DA were performed SIMCA-P + (Version 17.0; Umetrics, Umeå, Sweden) to assess metabolic differences between species. The PLS-DA model was validated by 100 × permutation test.

## Results and discussion

### Sugars and organic acids

Sucrose was identified as the predominant sugar constituent in both species, with concentration ranging from 17.89 g to 32.91 g/100 g in Korean ginseng and from 16.04 to 29.06 g/100 g in American ginseng, depending on the tissue part (Supplementary Table 2). In the whole root, sucrose content differed significantly between the two species ($p<$ 0.01) (Table 1). Overall, Korean ginseng had higher levels of sucrose. For example, in the body, Korean ginseng contained more than double the amount of sucrose (Supplementary Table 2). The predominance of sucrose is consistent with previous metabolomic studies identifying sucrose as the main primary metabolite in ginseng roots (Yoon et al., 2019) and one of the key discriminant metabolites distinguishing Korean and American ginseng (Lee et al., 2009). These findings collectively suggest that sucrose plays a central role in ginseng carbohydrate metabolism and serves as distinguishing metabolic feature between the two species.

**Table 1:** Metabolites profiles of Korean and American ginseng whole roots: sugars, organic acid, volatile compounds, and ginsenosides

	Korean ginseng	American ginseng	Significance
Sugars (g/100g)			
Sucrose	32.91 ± 3.26^a^	29.06 ± 1.65^b^	**
Organic acids (g/100g)			
Oxalic acid	0.26 ± 0.45^b^	0.86 ± 0.48^a^	*
Tartaric acid	4.58 ± 6.61^b^	22.44 ± 4.27^a^	***
Acetic acid	5.05 ± 1.52^a^	3.76 ± 0.97^b^	*
Citric acid	39.14 ± 27.32^a^	0.86 ± 0.30^b^	***
Succinic acid	6.47 ± 5.10^b^	20.12 ± 7.72^a^	***
Volatiles (μg/kg)			
Alcohols			
Ethanol	0.06 ± 0.08^a^	nd^b^	*
Alkenes			
cis-3-Undecene-1,5-diyne	0.19 ± 0.23^a^	nd^b^	*
Terpenes			
3-Carene	nd^b^	0.12 ± 0.11^a^	**
*α*-Guaiene	0.10 ± 0.14^a^	nd^b^	*
Bicyclogermacrene	0.38 ± 0.30^a^	nd^b^	***
*β*-Myrcene	0.11 ± 0.10^a^	0.02 ± 0.05^b^	*
*β*-Neoclovene	0.61 ± 0.53^a^	0.03 ± 0.06^b^	**
*β*-Selinene	0.19 ± 0.21^a^	nd^b^	**
Caryophyllene	1.12 ± 0.82^a^	nd^b^	***
*γ*-Maaliene	nd	0.26 ± 0.26	**
*γ*-Neoclovene	0.83 ± 1.24^a^	nd^b^	*
Ginsenol	0.04 ± 0.05^a^	nd^b^	*
Humulene oxide II	0.28 ± 0.27^a^	nd^b^	**
Modephene	0.24 ± 0.31^a^	nd^b^	*
Neointermedeol	0.06 ± 0.08^a^	nd^b^	*
Panaginsene	0.44 ± 0.49^a^	0.06 ± 0.10^b^	*
Panaxene	0.22 ± 0.26^a^	nd^b^	*
Ethers			
Carvacryl methyl ether	0.15 ± 0.21^a^	nd^b^	*
Others			
Methyl palmitate	0.03 ± 0.04^a^	nd^b^	**
Ginsenosides (normalized abundance)			
Ginsenoside Ra2	9,506.82 ± 8,212.6^a^	nd^b^	**
Ginsenoside Rb1	128.53 ± 266.58^b^	1,651.85 ± 439.82^a^	***
Notoginsenoside R4	3,203.02 ± 2,872.04^a^	84.98 ± 48.58^b^	**
Pseudoginsenoside RT1	984.81 ± 1028.33^b^	8,282.28 ± 3,932.59^a^	***
Pseudoginsenoside-F11	50.61 ± 167.87^b^	867.33 ± 893.88^a^	**
Quinquenoside R1	8,108.67 ± 13,688.89^b^	47,153.87 ± 10,143.37^a^	***

Different letters within a row indicate statistically significant differences. Significance levels: $p<$ 0.05 (*), $p<$ 0.01(**),$p<$ 0.001 (***)

Distinct differences were also observed in the profiles of organic acids between Korean and American ginseng (Table 1; Supplementary Table 2). The predominant organic acids detected in both species were malic, citric, succinic, tartaric and acetic acids, whereas oxalic and lactic acids were present at relatively low concentrations. Among these, for the whole sample, acetic acid ($p<$ 0.05) and citric acid ($p<$ 0.001) were significantly higher in Korean ginseng, while oxalic acid ($p<$ 0.05), tartaric acid ($p<$ 0.001), and succinic acid ($p<$ 0.001) were more abundant in American ginseng ($p<$ 0.05). While, malic acid accounted for the largest proportion of total organic acids in both species, no significant difference was observed.

The higher levels of citric and acetic acid in Korean ginseng may indicate greater tricarboxylic acid (TCA) cycle turnover and enhanced oxidative metabolism, consistent with active energy production and biosynthetic precursor supply. Citric acid has previously been identified as a discriminant metabolite reflecting ginseng age and metabolic activity (Park et al., 2013). Conversely, the elevated succinic acid content in American ginseng suggests increased flux through the latter stages of the TCA cycle, reflecting altered energy metabolism and adaptive carbon utilization. Similar patterns were reported in previous ginseng research, indicating that elevated citric acid and succinic acid concentrations were associated with enhanced energy metabolism and stress-responsive activity in ginseng roots (Chen et al., 2018). As key intermediates of the TCA cycle, succinic acid and malic acid are closely linked to respiratory activity and energy metabolism, and their elevated levels have been associated with enhanced metabolic energy flow in ginseng (Nguyen et al., 2016). Collectively, these findings suggest that Korean ginseng maintains a more oxidative metabolic profile with TCA cycle turnover, whereas American ginseng shows accumulation of downstream organic acids, indicating a shift toward carbon conservation.

### Volatile compounds

Headspace SPME-GC/MS profiling identified diverse volatile classes in both Korean and American ginseng, with terpenes representing the most abundant group (Supplementary Table 3; Supplementary Fig. 1). Consistent with previous finding, terpene-derived volatiles were predominant across all samples (Cho et al., 2012; Kim et al., 2022). Among plant parts, roots contained the highest concentrations of volatile in both species, indicating that the root tissue serves as the major reservoir of aroma-active compounds.

**Fig. 1:**
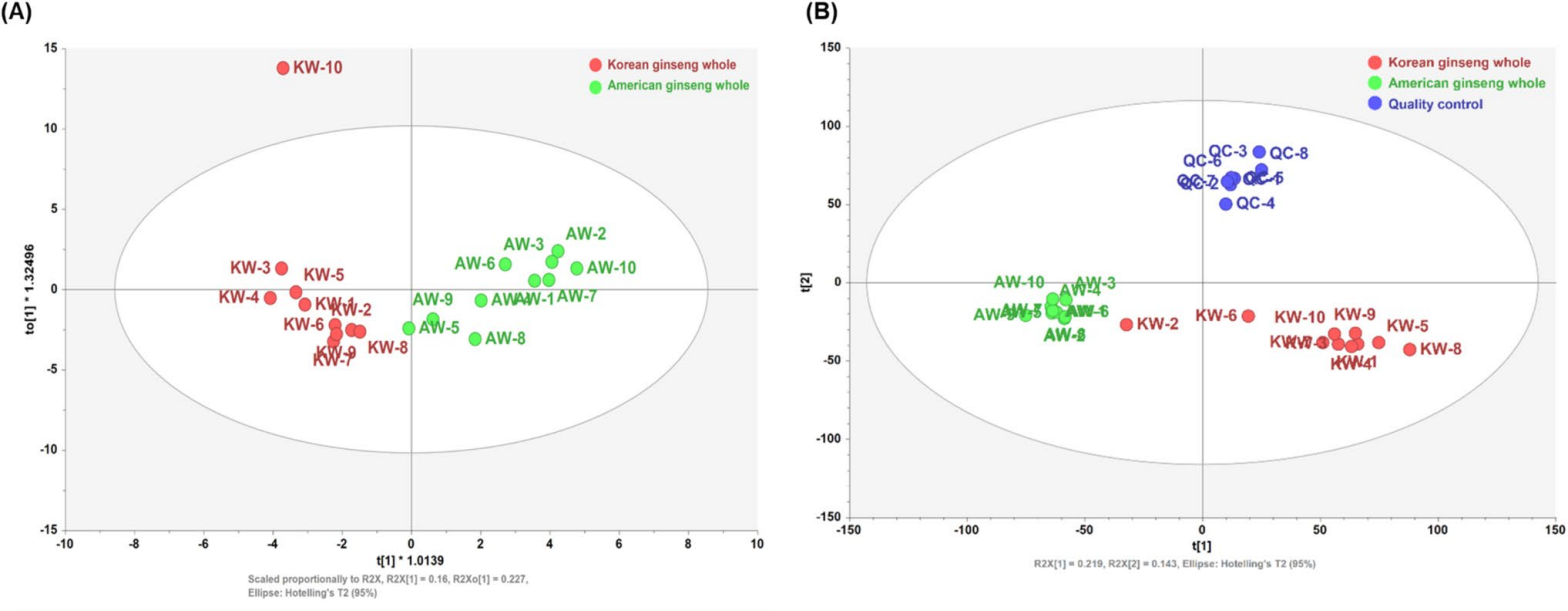
(**A**) OPLS-DA score plot based on volatile metabolite profiles, and (**B**) PLS-DA score plot derived from untargeted metabolomics data of Korean and American ginseng whole roots

Orthogonal partial least squares discriminant analysis (OPLS-DA) demonstrated clear separation between Korean and American ginseng based on volatile profiles across all tissues (whole, body, and root) (Fig. 1A; Supplementary Fig. 2). The OPLS-DA models also distinguished samples by plant part, explaining 27.8%–41.2% of the predictive variance and 8.9–13.9% of the orthogonal variance. Permutation testing confirmed model reliability (R^2^ = 0.46–0.53), indicating statistically valid group separation.

S-line and S-plot analyses identified discriminant volatiles contributing to interspecies differentiation. In whole roots, 2,2-dimethyl-3-hydroxypropionaldehyde and *β*-farnesene were enriched in American ginseng, whereas *α*-gurjunene, *β*-panasinsene, and *γ*-elemene were more abundant in Korean ginseng. In body tissues, American ginseng again showed elevated *β*-farnesene, while Korean ginseng contained higher levels of 1-butanol, *γ*-maaliene, *β*-panasinsene, and *γ*-neoclovene. In the root tissues, American ginseng exhibited greater amounts of octanal and *β*-farnesene, whereas Korean ginseng was enriched in ginsinsene, *γ*-maaliene, *β*-panasinsene, and *γ*-patchoulene.

These differences reflect divergence in the cyclization of the farnesyl diphosphate (FPP) pool through germacradienyl- or bisabolyl-type cation intermediates, which drive the structural diversification of ginseng sesquiterpenes (Cho et al., 2012). *β*-farnesene, an acyclic sesquiterpene derived from FPP, was abundant in American ginseng and its enrichment likely results from enhanced formation of bisabolyl-type intermediates during FPP cyclization. This compound has also been identified as a characteristic volatile of American ginseng, supporting its role as a species-specific marker (Cho et al., 2012). Furthermore, ginsinsene has been reported as one of the major sesquiterpenes isolated from *P. ginseng* roots, supporting its biochemical relevance as a maker compound (Richter et al., 2005).

Overall, terpene-derived volatile profiles were both tissue-specific and species-dependent. Among these, *β*-panasinsene (in Korean ginseng) and *β*-farnesene (in American ginseng) consistently emerged as robust discriminant markers, corroborating previous reports that identified these sesquiterpenes as characteristic indicators differentiating Korean and American ginseng (Cho et al., 2012; Kim et al., 2022). *β*-Farnesene is known for its sweet aroma quality (Cui et al., 2015). The aroma-active properties of ginseng-specific sesquiterpenes such as *β*-panasinsene have not yet been characterized, and their sensory contributions remain to be investigated (Kim et al., 2022).

### Untargeted metabolomics

Untargeted metabolomics using UPLC-QTOF/MS revealed distinct metabolic profiles between Korean and American ginseng. A total of 2,601 and 11,268 metabolites ($p\le$ 0.05) were detected in positive and negative ionization modes, respectively. Distinct chromatographic patterns were observed between the two species (Supplementary Fig. 3), indicating broad compositional differences in their metabolites.

Partial least squares discriminant analysis (PLS-DA) confirmed robust species-level separation (Fig. 1B), with PC1 and PC2 explaining 21.9% and 14.3% of the variance, respectively. Model reliability was supported by Y-prediction (RMSE = 0.219), and permutation testing (R^2^ = 0.838, Q^2^ = −0.00081).

S-plot and S-line analyses identified key discriminant metabolites differentiating the two species (Supplementary Fig. 4). In the S-plot, ginsenoside Ra2 and notoginsenoside R4 were positively correlated with Korean ginseng, whereas quinquenoside R1 was positively correlated with American ginseng, indicating clear species-specific associations. Quantitative analysis of representative ginsenosides further supported these results (Table 1) and largely agreed with previous comparative studies (Chen et al., 2008, 2020). Ginsenoside Ra2 and notoginsenoside R4 were significantly higher in Korean ginseng, with ginsenoside Ra2 detected exclusively in Korean ginseng ($p<$ 0.01). In contrast, ginsenoside Rb1, pseudoginsenoside RT1, and quinquenoside R1 were markedly higher in American ginseng ($p<$ 0.001). Notably, ginsenoside Ra2 was reported as a distinguishing marker for Korean ginseng (Kim et al., 2024), consistent with the current finding. Although quinquenoside R1 was detected in both species, its high variable importance in the S-plot and substantially higher abundance in American ginseng ($p<$ 0.001) suggest that it may serve as a potential indicator of this species. Higher levels of quinquenoside R1 in American ginseng have also been reported in a previous comparative study (Chen et al., 2008).

In addition to ginsenosides, terpenoid-related metabolites also differed between the two species (Supplementary Fig. 5). Korean ginseng exhibited higher levels of triterpenoids such as ganosinesin A and fasciculic acid A, as well as sesquiterpenes including glaciapyrrole C, sespendole, along with triterpenes such as inoterpene F. Only one triterpene compound, Pitheduloside K, was more abundant in American ginseng. As shown in Supplementary Fig. 5, the overall abundance of triterpenes and sesquiterpenes was substantially higher in Korean ginseng, highlighting these compounds as potential biomarker for distinguishing Korean and American ginseng. Triterpenes and sesquiterpenes are known to function as defense-related metabolites in plants, playing roles in antimicrobial activity and stress responses of ginsengs (Yoon et al., 2019). The higher abundance of these compounds in Korean ginseng may reflect species-specific variation in terpenoid biosynthesis.

### Electronic tongue analysis

**Fig. 2:**
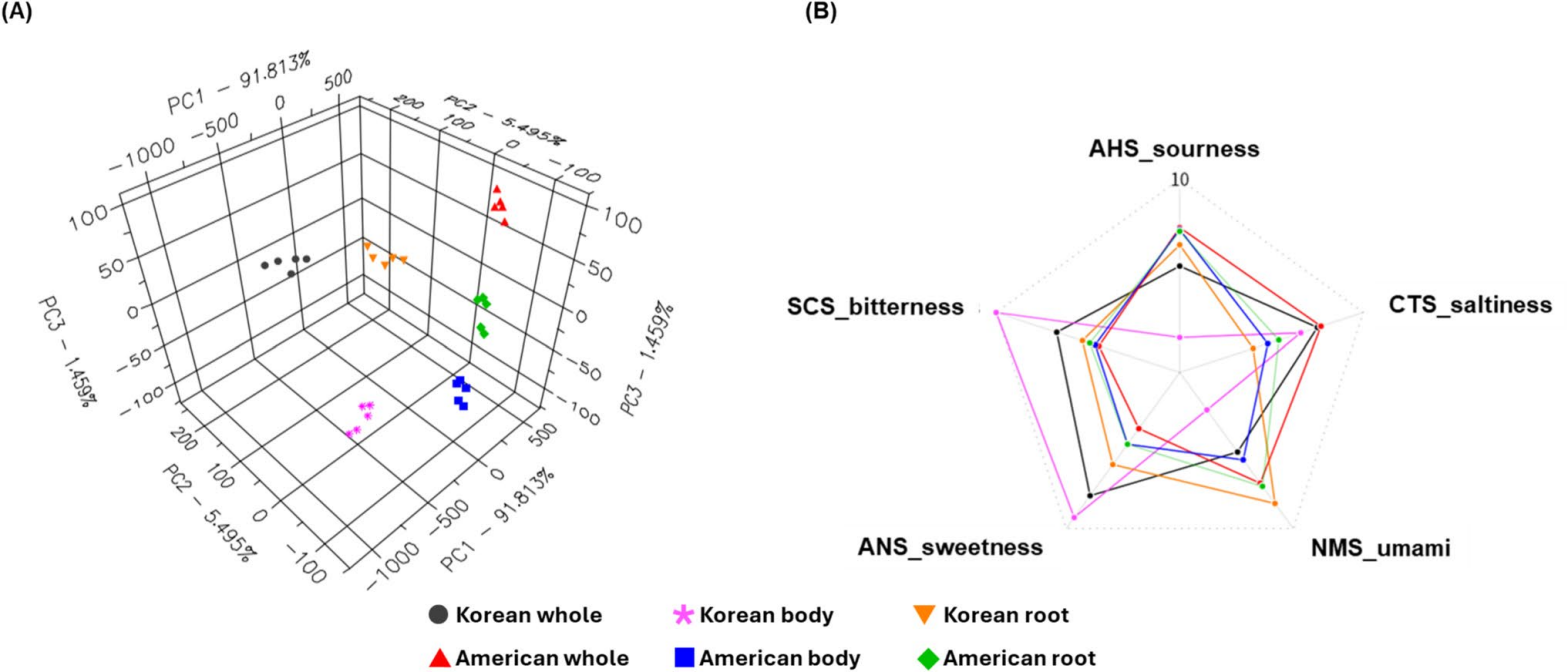
(**A**) PCA score scatter plots showing sensory variation between Korean and American ginseng across different plant parts (whole, body, and root) based on E-tongue sensor data. (**B**) Radar plot illustrating E-tongue taste profiles of Korean and American ginseng*.* The five sensory attributes—sourness (AHS), bitterness (SCS), sweetness (ANS), saltiness (CTS), and umami (NMS)—were evaluated for the whole, body, and root parts of each species

Electronic tongue profiling revealed distinct differences in taste attributes between Korean and American ginseng (Fig. 2; Supplementary Table 4). Sourness (anion-sensitive hydrogen; AHS sensor) was higher in American ginseng across all plant parts. Although Korean ginseng contained higher acetic acid and citric acid, this discrepancy may reflect matrix buffering effects that reduce free proton activity, resulting in lower AHS responses despite higher organic acid concentrations. The AHS sensor signal is determined by proton dissociation rather than total acid abundance (Dong et al., 2017). Moreover, previous sensory evaluation reported that sourness contributed minimally to interspecies discrimination among *Panax* species, whereas bitterness and sweetness are more obvious and influential (Cui et al., 2015).

Korean ginseng exhibited stronger bitterness (SCS sensor), reflecting its higher ginsenoside concentration, including ginsenoside Ra2 and notoginsenoside R4. This trend aligns with previous finding that Korean ginseng typically shows greater bitterness and after-bitterness intensities than American ginseng due to its higher ginsenoside concentration (Cui et al., 2015). The high levels of terpenoid-related metabolites such as ganosinesin A and fasciculic acid A in Korean ginseng might also contribute to its increased bitterness (Supplementary Fig. 5). Sweetness (ANS sensor) was also more pronounced in Korean ginseng, in agreement with the higher sucrose levels observed in Sect. 3.1 and with the established response of the ANS sensor to sugar-derived potentials (Dong et al., 2017). Meanwhile, saltiness (CTS) and umami (NMS) responses showed no significant differences between species. This pattern agrees with earlier observation that ionic and amino acid-related tastes contributed less to ginseng species discrimination (Tian et al., 2021).

Collectively, these results demonstrate that Korean ginseng is characterized by a sweeter and more bitter profile driven by its higher sucrose, terpenoids, and ginsenoside contents, whereas American ginseng exhibits a relatively sour taste associated with organic acid accumulation, which is expected to contribute to a milder, less harsh flavor. These trends align with compositional differences in sugars, organic acids, and ginsenosides described in previous sections, confirming that metabolic divergence translates into taste characteristics. (Cui et al., 2015; Dong et al., 2017; Tian et al., 2021). Both species were cultivated under identical conditions, enabling a controlled comparison; however, validation across different harvest years would further confirm the robustness of the identified markers. Additionally, incorporating trained sensory panels in future studies could complement the e-tongue data by capturing more comprehensive flavor characteristics.

This study employed a multi-platform metabolomics approach to discriminate Korean and American ginseng across different tissues. Distinct interspecies differences were identified sugars, organic acids, volatile compounds, untargeted metabolite profiles, and taste attributes. Korean ginseng was enriched in sucrose, acetic acid, citric acid, *β*-panasinsene, ginsenoside Ra2, and other terpenoid derivatives, whereas American ginseng contained higher levels of succinic acid, tartaric acid, *β*-farnesene, and quinquenoside R1. Electronic tongue analysis further supported this chemical differentiation, revealing stronger bitterness and sweetness in Korean ginseng and higher sourness in American. Overall, this integrative metabolomics framework provides a reliable chemical and sensory basis for ginseng authentication, offering potential applications in quality control, regulatory monitoring, and functional food development.

## Supplementary Information

Below is the link to the electronic supplementary material.

Supplementary file1 (DOCX 1253 KB)
