# Supplementary material for: Chemical fingerprinting of Korean ginseng (Panax ginseng) and American ginseng (Panax quinquefolius) using multi-platform metabolomics and taste profiling
Source: Food Sci Biotechnol. 2026 Apr 8;35(6):1459–66. doi: 10.1007/s10068-026-02137-5 (PMC13129142; doi:10.1007/s10068-026-02137-5)
Supplement: Supplementary file 1 — Supplementary file1 (DOCX 1253 KB) [file 10068_2026_2137_MOESM1_ESM.docx]

**Supplementary Materials**

**Chemical fingerprinting of Korean ginseng (*Panax ginseng*) and American ginseng (*Panax quinquefolius*) using multi-platform metabolomics and taste profiling**

**Running Title: Chemical Fingerprinting of Ginseng Species**

Namhee Lee^1,#^, Jung-Woo Lee^2,#^, Yejin Kim^3^, So-Jeong Kim^2^, Kyong-Hwan Bang^2^, Unyong Kim^4^, Sang Beom Han^4^, Jeehye Sung^3,*^, Joon Hyuk Suh^1,*^

*^1^Department of Food Science and Technology, College of Agricultural and Environmental Sciences, University of Georgia, 100 Cedar Street, Athens, GA 30602, USA*

*^2^Department of Herbal Crop Research, National Institution of Horticultural and Herbal Science, Rural Development Administration, Eumseong 27709, Republic of Korea*

*^3^Department of Food Science and Biotechnology, Gyeongkuk National University, Andong, 36729, South Korea*

*^4^Department of Pharmaceutical Analysis, College of Pharmacy, Chung-Ang University, 84 Heukseok-Ro, Dongjak-Gu, Seoul 06974, Republic of Korea*

^#^These authors contributed equally to this work.

^*^Co-corresponding authors.

Corresponding authors:

Jeehye Sung, Ph.D.

Email: [jeehye@gknu.ac.kr](mailto:jeehye@gknu.ac.kr); Telephone: +82-54-820-5752

Address: 1375 Gyeongdong-ro, Andong-si, Gyeongsangbuk-do, 36729, Korea

Joon Hyuk Suh, Ph.D.

Email: [J.Suh@uga.edu](mailto:J.Suh@uga.edu); Telephone: +1-706-542-7907

Address: 100 Cedar Street, Athens, GA 30602, USA

**Chemicals and reagents**

Sucrose, malic acid, acetic acid, citric acid, succinic acid, tartaric acid, oxalic acid, and 2-methylbutyl 2-methylbutyrate was purchased from Sigma-Aldrich (St. Louis, MO, USA). All other chemicals were of analytical grade.

**Free sugar analysis**

Freeze-dried ginseng powder (0.1 g) was extracted with 5 mL of 50% acetonitrile, a solvent concentration chosen to efficiently extract mono- and disaccharides while minimizing the co-extraction of hydrophobic constituents for optimal compatibility with an NH_2_-type column chromatography (Sławińska et al., 2021). Following centrifugation at 3,000 g for 15 min, the supernatants were filtered through a 0.45 µm nylon membrane filter (Whatman, Clifton, NJ, USA). Chromatographic separation was performed using an HPLC system equipped with an Asahipak NH2P-50 column (4.6 × 250 mm, 5 µm; Shodex, Tokyo, Japan) and a refractive index detector (Waters, Milford, MA, USA). The mobile phase consisted of acetonitrile and water (75:25, v/v) at a flow rate of 1.0 mL/min, with an injection volume of 10 µL under isocratic elution conditions.

**Organic acid analysis**

Freeze-dried ginseng powder (0.03 g) was extracted with 1 mL of 0.2% phosphoric acid, vortexed for 5 min, and subsequently sonicated for 30 min at 4°C. Aqueous 0.2% phosphoric acid was specifically employed to facilitate the solubilization of organic acids and maintain compatibility with the acidic mobile phase, thereby improving chromatographic resolution on the C18 column (Agius et al., 2018). After extraction, samples were centrifuged at 12,000 rpm for 20 min at 4℃, and filtered through a 0.2 µm nylon membrane filter. The supernatants were analyzed using an HPLC system equipped with a UV detector (Jasco UV-2075, Tokyo, Japan). Chromatographic separation was achieved on a Cadenza CD-C18 column (4.6 $\times$ 250 mm, 3 µm) with a mobile phase consisting of (A) 0.2% phosphoric acid in water and (B) acetonitrile. The gradient elution was carried out as follows: 0-30 min, 0% B; 30-37 min, 30% B; 37-39 min, 30% B; 39-40 min, 0% B; 40-50 min, 0% B. The column temperature was set at 30℃, the flow rate was 0.6 mL/min, injection volume was 10 µL, and detection wavelength was 220 nm. Quantification was based on calibration curves of authentic standards.

**Volatile compound analysis**

Volatile compounds of ginseng samples were analyzed using headspace solid-phase microextraction coupled with gas chromatography-mass spectrometry (HS-SPME-GC/MS). Freeze-dried ginseng powder (2 g) was placed in a 20 mL SPME vial containing 3 mL of distilled water and 10 µg/mL of 2-methylbutyl-2-methylbutyrate as an internal standard. Extraction was performed using a 65 µm divinylbenzene/polydimethylsiloxane (DVB/PDMS) fiber (Supelco, Bellefonte, PA, USA), which was selected for its high sensitivity toward a broad range of ginseng volatiles (Woyciechowski et al., 2026). The ginseng sample was stirred continuously at 250 rpm and 50 °C for 40 min, during which the SPME fiber was exposed to the headspace for 20 min under the same conditions.

The analysis was performed using a GC/MS system (QP2020, Shimadzu, Kyoto, Japan) equipped with an HP-FFAP capillary column (50 m × 0.32 mm i.d., 0.65 μm film thickness; Agilent Technologies, Wilmington, DE). Helium was used as the carrier gas at a constant flow rate of 1.0 mL/min. The injector temperature was set at 230℃, and mass spectra were obtained in electron impact (EI) mode at 70 eV over the range of m/z 30-500. The oven temperature program was as follows: initial temperature of 50℃ held for 2 min, increased to 80℃ at 5℃/min and held for 3 min, then ramped to 250℃ at 3℃/min and maintained for 5 min. Volatile compounds were identified by comparing mass spectra with the NIST14 library (National Institute of Standards and Technology, Gaithersburg, MD, USA) and matching retention indices (RI), calculated using an n-alkane series (C7–C30), with literature values. Semi-quantitative analysis was performed by calculating the peak area ratio of each analyte to the internal standard.

**Untargeted metabolomics (UPLC-QTOF/MS)**

Freeze-dried ginseng powder (0.05 g) was mixed with 1 mL of an acetonitrile/isopropanol/water mixture (3:3:2, v/v/v), a solvent system specifically chosen to cover a wide polarity range for the comprehensive and simultaneous extraction of diverse metabolite classes (Cajka and Fiehn, 2016; Wang et al., 2026). The mixture was vortexed for 5 min and subsequently sonicated for 30 min at 4°C. Following extraction, the samples were centrifuged at 12,000 g for 20 min at 4°C and filtered through a 0.2 µm nylon membrane filter. The supernatants were analyzed using a Waters SYNAPT G2-Si Q-TOF MS coupled to an ACQUITY UPLC I-Class system (Waters, Milford, MA, USA). Separation was performed on an Acclaim C30 column (2.1 $\times$ 150 mm, 3 µm; Thermo Scientific) at 30℃. The mobile phases were (A) 0.1% formic acid in water and (B) 0.1% formic acid in acetonitrile. The gradient elution was: 0–3 min, 2% B; 3–25 min, 50% B; 25–30 min, 90% B; 30–30.1 min, 95% B; 30.1–35 min, 95% B; and 35.1–45 min, 2% B. The flow rate was 0.2 mL/min, and the mass range was m/z 50-1,200. The IMS condition is described in Supplementary Table 1. Data were acquired in positive and negative ion modes using MS^E^ acquisition, with leucine enkephalin as the lock mass. Progenesis QI (Nonlinear Dynamics, Newcastle, UK) was used for data processing, peak alignment, and metabolite annotation.

**Electronic tongue analysis**

Freeze-dried ginseng powder (3 g) was homogenized with 150 mL distilled water, sonicated for 20 min at 4℃, and filtered. The filtrate (25 mL) was used for electronic tongue analysis using an Astree Ⅱ system (Alpha MOS, Toulouse, France) equipped with seven sensors (AHS, PKS, CTS, NMS, CPS, ANS, and SCS) representing sourness, saltiness, umami, sweetness, and bitterness, with PKS and CPS serving as reference. Measurements were carried out at 25℃ with sensing time of 120s for each sample. Each sample was analyzed in five replicates. The response signals were recorded and processed using Alphasoft V 12.4 and Arochembase V4 (Alpha MOS).

**Supplementary Table 1.** Cyclic IMS condition

| **TOF setting** | |
| --- | --- |
| **Positive mode** | **Negative mode** |
| Run time: 45min  Analyser mode: V-mode  Mass range: 50-1200  MS mode: MSe  Cone voltage: 25  Collision Energy: 10-45  Scan time: 0.1s  Lock Spray: Leucine Enkephalin  Lock Spray interval: 5min | Run time: 45min  Analyser mode: V-mode  Mass range: 50-1200  MS mode: MSe  Cone voltage: 25  Collision Energy: 10-45  Scan time: 0.1s  Lock Spray: Leucine Enkephalin  Lock Spray interval: 5min |
| **Cyclic setting** | |
| **Positive mode** | **Negative mode** |
| TW staic height: 22.0V  Inject: 10ms  Separate: 5ms  Eject and Acquire:13.2ms | TW staic height: 22.0V  Inject: 10ms  Separate: 5ms  Eject and Acquire:13.2ms |

**Supplementary Table 2.** Sucrose and organic acid contents of Korean and American ginseng (g/100g)

| **Sample** | | | **Sucrose** | **Organic acid** | | | | | | |
| --- | --- | --- | --- | --- | --- | --- | --- | --- | --- | --- |
|  | | |  | **Oxalic acid** | **Tartaric acid** | **Malic acid** | **Lactic acid** | **Acetic acid** | **Citric acid** | **Succinic acid** |
| Korean ginseng | Whole | 32.91 ± 3.26^a^ | | 0.26 ± 0.45^a^ | 4.58 ± 6.61^c^ | 47.80 ± 10.95^ab^ | nd^a^ | 5.05 ± 1.52^ab^ | 39.14 ± 27.32^b^ | 6.47 ± 5.10^b^ |
|  | Body | 33.46 ± 2.81^a^ | | nd^ab^ | 2.12 ± 1.14^c^ | 45.72 ± 9.58^ab^ | nd^a^ | 5.69 ± 1.86^ab^ | 42.26 ± 25.36^b^ | 14.11 ± 3.11^b^ |
|  | Root | 17.89 ± 1.81^ab^ | | 3.43 ± 3.08^b^ | 2.84 ± 0.85^c^ | 57.88 ± 8.40^b^ | 0.67 ± 0.73^b^ | 8.39± 6.91^b^ | 28.20 ± 17.89^b^ | 18.60 ± 9.00^ab^ |
| American ginseng | Whole | 29.06 ± 1.65^b^ | | 0.86 ± 0.48^ab^ | 22.44 ± 4.27^a^ | 56.16 ± 9.82^ab^ | nd | 3.76 ± 0.97^ab^ | 0.86 ± 0.30^a^ | 20.12 ± 7.72^ab^ |
|  | Body | 16.04 ± 1.26^bc^ | | 1.95 ± 1.75^b^ | 2.53 ± 0.96^b^ | 43.69 ± 13.46^a^ | nd | 1.57 ± 1.15^a^ | 17.89 ± 12.18^ab^ | 9.52 ± 4.70^a^ |
|  | Root | 24.37 ± 2.70^c^ | | 1.16 ± 0.84^ab^ | 16.24 ± 8.21^c^ | 67.93 ± 5.89^c^ | nd | 4.65 ± 2.57^ab^ | 14.28 ± 15.65^ab^ | 35.55 ± 11.86^c^ |

Different letters within a column indicate statistically significant differences ($p<$ 0.05).

**Supplementary Table 3.** Profiles of volatile compounds in Korean and American ginseng *(*μg/kg)

| **Compound** | **RT^A^** | **RI^B^** | **Korean ginseng** | | | **American ginseng** | | | **Identification^C^** |
| --- | --- | --- | --- | --- | --- | --- | --- | --- | --- |
|  |  |  | **Whole** | **Body** | **Root** | **Whole** | **Body** | **Root** |  |
| ***Acids*** | | | | | | | | | |
| Hexanoic acid | 46.46 | 1777 | 0.04 | 0.38 | nd | 0.06 | 0.07 | 0.12 | MS, TI |
| Octanoic acid | 50.2 | 1884 | 0.07 | 0.44 | 0.61 | 0.46 | 0.06 | 0.21 | MS, TI |
| ***Alcohols*** | | | | | | | | | |
| Ethanol | 9.98 | 852 | 0.04 | 0.94 | 0.18 | 0.62 | 0.10 | 0.38 | MS, TI |
| 1-Butanol | 16.51 | 1061 | nd | 5.12 | 0.48 | 0.04 | nd | 0.19 | MS, TI |
| 2-Heptanol | 24.16 | 1236 | 0.08 | 0.21 | 1.01 | 0.07 | nd | 0.03 | MS, TI |
| 1-Hexanol | 25.85 | 1273 | 0.37 | 0.80 | 1.39 | 0.89 | 0.09 | 0.24 | MS, TI, FI |
| 1-Heptanol | 30.46 | 1376 | 0.08 | 0.47 | 0.81 | 0.13 | 0.06 | 0.15 | MS, TI |
| 2,3-Butanediol | 34.74 | 1476 | 0.11 | 0.18 | 0.70 | 0.23 | nd | 0.13 | MS, TI |
| 1-Octanol | 34.89 | 1479 | 0.38 | 1.84 | 3.42 | 2.22 | 0.30 | 0.27 | MS, TI, FI |
| 1-Nonen-3-ol | 38.72 | 1573 | 0.01 | 0.04 | nd | 0.05 | nd | 0.08 | MS, TI |
| 2-Phenylethanol | 49.59 | 1866 | nd | nd | 0.48 | nd | nd | nd | MS, TI |
| Spathulenol | 56.55 | 2078 | 0.08 | 0.66 | 0.76 | nd | 0.01 | 0.01 | MS, TI |
| ***Aldehydes*** | | | | | | | | | |
| Hexanal | 14.26 | 1004 | 0.07 | 0.08 | nd | 1.02 | 0.05 | 0.41 | MS, TI |
| 2,2-Dimethyl-3-hydroxypropionaldehyde | 16.51 | 1061 | nd | nd | 1.17 | 7.45 | nd | nd | MS, TI |
| Octanal | 23.21 | 1215 | 0.20 | 0.04 | nd | 0.43 | 0.16 | 1.98 | MS, TI, FI |
| 2-Nonenal | 34.31 | 1466 | 0.02 | nd | nd | 0.06 | 0.05 | 0.20 | MS, TI |
| ***Alkenes*** | | | | | | | | | |
| 1-Undecene | 15.88 | 1045 | nd | 0.07 | 0.09 | 0.01 | 0.18 | 0.16 | MS, TI, FI |
| ***Etc*** | | | | | | | | | |
| Ginsenoyne E | 50.24 | 1885 | nd | 0.26 | 0.38 | nd | nd | nd | MS, TI |
| ***Ethers*** | | | | | | | | | |
| Methyl octanoate | 27.42 | 1308 | 0.01 | 0.13 | 0.04 | 0.24 | 0.01 | 0.16 | MS, TI |
| Carvacryl methyl ether | 36.43 | 1517 | nd | 0.15 | 1.01 | nd | nd | nd | MS, TI |
| ***Ketones*** | | | | | | | | | |
| Acetoin | 23.75 | 1227 | 0.11 | 0.22 | 0.51 | 0.25 | 0.02 | 0.09 | MS, TI |
| 2-Nonanone | 27.84 | 1317 | 0.02 | 0.08 | 0.14 | 0.19 | 0.01 | 0.08 | MS, TI, FI |
| ***Furnas*** | | | | | | | | | |
| 2-Pentyl furan | 20.27 | 1149 | 0.09 | 0.35 | 0.10 | 0.05 | 0.04 | 0.11 | MS, TI, FI |
| ***Terpenes*** | | | | | | | | | |
| α-Pinene | 12.03 | 934 | 0.16 | 0.08 | 1.44 | 0.21 | 0.19 | 0.51 | MS, TI, FI |
| Camphene | 13.49 | 981 | 0.08 | 0.01 | 0.46 | 0.06 | 0.03 | 0.23 | MS, TI |
| β-Pinene | 15.02 | 1023 | 0.16 | 0.46 | 0.64 | 0.15 | 0.09 | 0.63 | MS, TI, FI |
| 3-Carene | 17.31 | 1081 | nd | nd | nd | 0.12 | 0.12 | 0.19 | MS, TI |
| β-Myrcene | 17.39 | 1083 | 0.11 | 0.05 | 0.65 | 0.02 | 0.05 | 0.04 | MS, TI |
| D-Limonene | 19.12 | 1124 | 0.05 | nd | 0.07 | 0.07 | 0.04 | 0.32 | MS, TI |
| Modephene | 27.8 | 1316 | 0.24 | 0.60 | 0.54 | nd | nd | nd | MS, TI |
| Panaginsene | 28.57 | 1334 | 0.44 | 1.11 | 0.02 | 0.06 | 0.06 | 0.27 | MS, TI |
| Panaxene | 28.86 | 1340 | 0.22 | 0.25 | 1.49 | nd | 0.02 | 0.02 | MS, TI |
| δ-Selinene | 29.45 | 1354 | 0.08 | nd | 2.41 | nd | nd | nd | MS, TI |
| Ginsinsene | 29.89 | 1364 | 0.81 | 2.34 | 10.13 | 0.39 | 0.12 | 0.41 | MS, TI |
| α-Gurjunene | 30.11 | 1368 | 1.20 | 0.50 | 3.90 | nd | 0.04 | nd | MS, TI |
| Aristolene | 30.17 | 1370 | nd | 0.18 | 1.93 | 0.03 | 0.05 | 0.08 | MS, TI |
| β-Maaliene | 30.84 | 1385 | 0.58 | 0.52 | 1.33 | nd | 0.09 | 0.04 | MS, TI |
| α-Isocomene | 31.52 | 1400 | 0.50 | nd | 2.54 | 0.01 | nd | nd | MS, TI |
| Copaene | 31.57 | 1401 | 0.03 | nd | nd | nd | 0.04 | 0.20 | MS, TI |
| α-Cubebene | 31.59 | 1402 | nd | nd | 0.04 | 0.68 | nd | nd | MS, TI |
| alpha-Guaiene | 32.7 | 1428 | 0.10 | 0.90 | 3.96 | nd | 0.01 | 0.25 | MS, TI, FI |
| Cedrene-V6 | 32.93 | 1433 | 0.12 | 0.11 | 4.25 | 0.03 | nd | nd | MS, TI |
| γ-Maaliene | 33.01 | 1435 | nd | 9.35 | 9.59 | 0.26 | 0.60 | nd | MS, TI |
| Aromandendrene | 33.04 | 1436 | 0.47 | nd | nd | nd | 0.30 | nd | MS, TI |
| Valencene | 33.68 | 1451 | nd | nd | 1.88 | 1.88 | nd | nd | MS, TI |
| β-Panasinsene | 34.3 | 1466 | 2.44 | 5.60 | 20.44 | 0.11 | 0.32 | 1.62 | MS, TI |
| δ-Panasinsine | 35.55 | 1495 | 0.04 | 0.27 | nd | nd | nd | nd | MS, TI |
| Alloaromadendrene | 36.09 | 1508 | nd | 0.40 | 7.69 | nd | 0.02 | nd | MS, TI |
| Calarene | 37.11 | 1533 | 0.90 | 0.11 | nd | nd | nd | 0.77 | MS, TI |
| α-Panasinsen | 37.16 | 1535 | nd | 0.28 | nd | nd | 0.01 | 0.16 | MS, TI |
| Valerena-4,7(11)-diene | 37.75 | 1549 | 1.64 | 4.86 | 7.27 | nd | 0.15 | 0.13 | MS, TI |
| γ-Patchoulene | 38.24 | 1561 | nd | nd | 10.21 | nd | nd | nd | MS, TI |
| γ-Neoclovene | 39.11 | 1583 | nd | 9.89 | 3.70 | nd | 0.02 | 0.35 | MS, TI |
| β-Famesene | 39.25 | 1586 | 0.49 | 1.28 | 0.79 | 6.86 | 1.91 | 3.34 | MS, TI, FI |
| γ-Elemene | 39.33 | 1588 | 1.39 | 1.39 | 5.97 | nd | 0.10 | nd | MS, TI |
| α-Neoclovene | 39.51 | 1593 | 0.16 | 1.02 | nd | 0.18 | 0.10 | 0.35 | MS, TI, FI |
| Humulene | 40.06 | 1607 | 0.66 | 1.97 | 0.28 | 0.43 | 0.04 | 0.23 | MS, TI |
| β-Elemene | 40.19 | 1610 | 0.47 | 0.44 | 1.97 | 0.02 | 0.02 | 0.06 | MS, TI, FI |
| Caryophyllene | 40.33 | 1614 | 1.18 | 2.59 | 6.01 | nd | nd | 1.30 | MS, TI, FI |
| β-Neoclovene | 41.34 | 1640 | 0.61 | 2.08 | 6.09 | 0.04 | nd | 0.18 | MS, TI |
| β-Selinene | 41.39 | 1641 | 0.19 | 0.76 | 2.76 | nd | 0.03 | 0.04 | MS, TI |
| α-Selinene | 42.24 | 1664 | nd | 0.41 | nd | nd | nd | 0.10 | MS, TI |
| γ-Selinene | 42.25 | 1664 | nd | nd | 1.71 | nd | nd | nd | MS, TI |
| γ-Elemene | 42.59 | 1673 | nd | 0.03 | nd | 1.03 | nd | nd | MS, TI |
| β-Cadinene | 42.8 | 1678 | 0.02 | nd | nd | 0.64 | 0.18 | 0.08 | MS, TI |
| β-Sesquiphellandrene | 43.31 | 1691 | nd | 0.17 | 0.21 | nd | 0.01 | nd | MS, TI, FI |
| Pacifigorgiol | 50.4 | 1890 | 0.28 | 0.92 | 3.25 | nd | nd | 0.04 | MS, TI |
| Humulene oxide II | 54.23 | 2005 | 0.03 | 0.64 | 0.41 | nd | nd | nd | MS, TI |
| Caryophyllene oxide | 55.49 | 2044 | 0.06 | 0.36 | 1.21 | nd | 0.01 | 0.03 | MS, TI, FI |
| Neointermedeol | 56.92 | 2089 | 0.04 | 0.17 | 1.04 | nd | 0.01 | 0.01 | MS, TI |
| Ginsenol | 57.28 | 2101 | 0.11 | 0.16 | 1.11 | nd | 0.01 | 0.01 | MS, TI |

^A^Retention time on the HP-FFAP capillary column; ^B^Retention indices were determined on HP-FFAP capillary column using n-alkanes (C7-C30) as external reference; ^C^Identification with FI (fully identified using authentic standard), MS (mass spectrum consistent with that from the NIST library), TI (tentatively identified based on the NIST library and literature); ^D^Not detected

**Supplementary Table 4.** Electronic tongue (e-tongue) sensor responses for Korean and American ginseng measured in whole, body, and root parts.

| **Sample names** | | **Sensors^a^** | | | | | | |
| --- | --- | --- | --- | --- | --- | --- | --- | --- |
|  |  | **AHS** | **PKS** | **CTS** | **NMS** | **CPS** | **ANS** | **SCS** |
| Korean ginseng | Whole | 4,127.59 | 28.91 | 419.24 | 1,638.77 | 1,470.78 | 3,367.97 | 4,499.39 |
|  | Root | 3,993.62 | -99.82 | 351.68 | 1,537.73 | 1,486.71 | 3,068.25 | 4,327.90 |
|  | Body | 4,563.83 | 94.51 | 403.08 | 1,721.10 | 1,826.01 | 3,581.02 | 4,937.16 |
| American ginseng | Whole | 3,884.78 | -210.95 | 422.28 | 1,576.21 | 1,453.60 | 2,737.85 | 4,206.25 |
|  | Root | 3,909.22 | -187.22 | 380.03 | 1,570.09 | 1,583.10 | 2,885.23 | 4,267.37 |
|  | Body | 3,890.50 | -67.66 | 368.12 | 1,622.84 | 1,596.51 | 2,882.13 | 4,230.21 |

^a^ AHS; sensor for sourness, PKS; sensor for reference, CTS; sensor for saltiness, NMS; sensor for umami, saltiness and astringency, CPS; sensor for standard, ANS; sensor for sweetness and sourness, SCS; sensor for bitterness and astringency


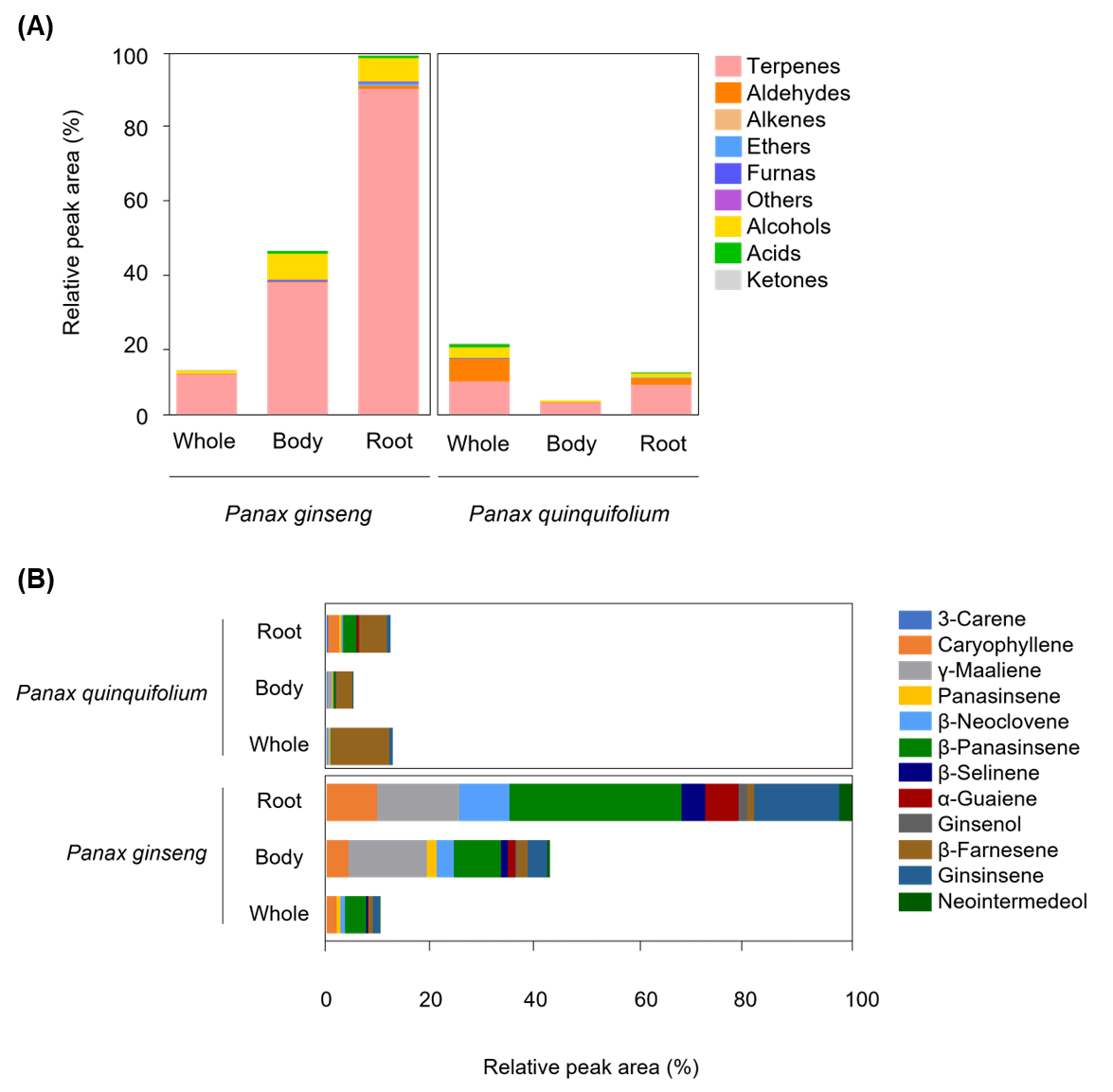


**Supplementary Figure 1.** Relative profiles of volatile compounds in Korean and American ginseng*.* (A) Overview of volatile compound distribution in Korean and American ginseng based on total peak percentages (100% = Korean ginseng root). (B) Profiles of major discriminant volatile compounds identified in whole, body, and root parts of Korean and American ginseng*.* Values represent mean relative abundances (n = 10 biological replicates).


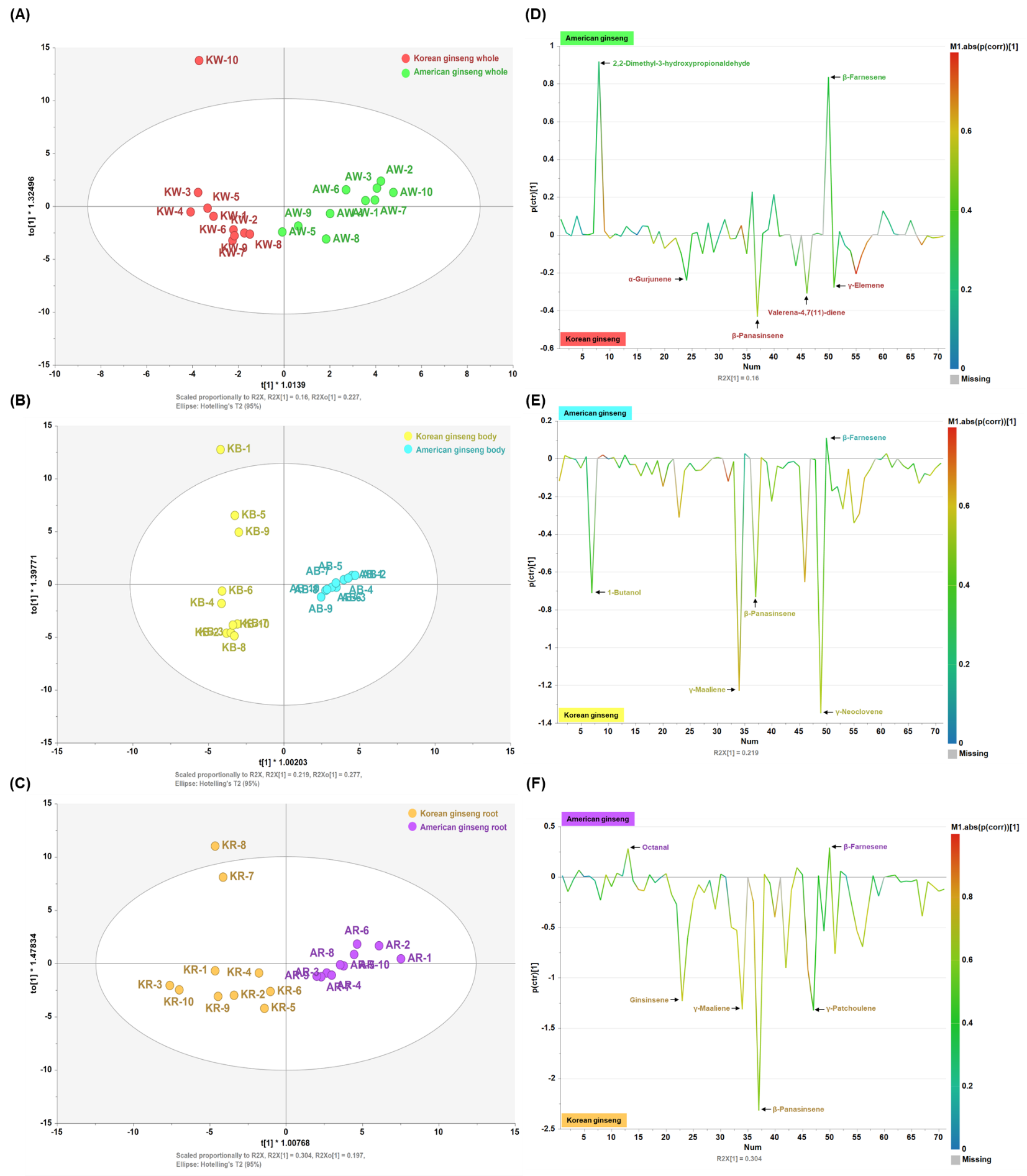


**Supplementary Figure 2.** OPLS-DA of volatile profiles in Korean and American ginseng. (A-C) PLS-DA score plots for (A) whole, (B) body, and (C) root samples. (D-F) Corresponding S-line plots highlighting variables driving separation

**
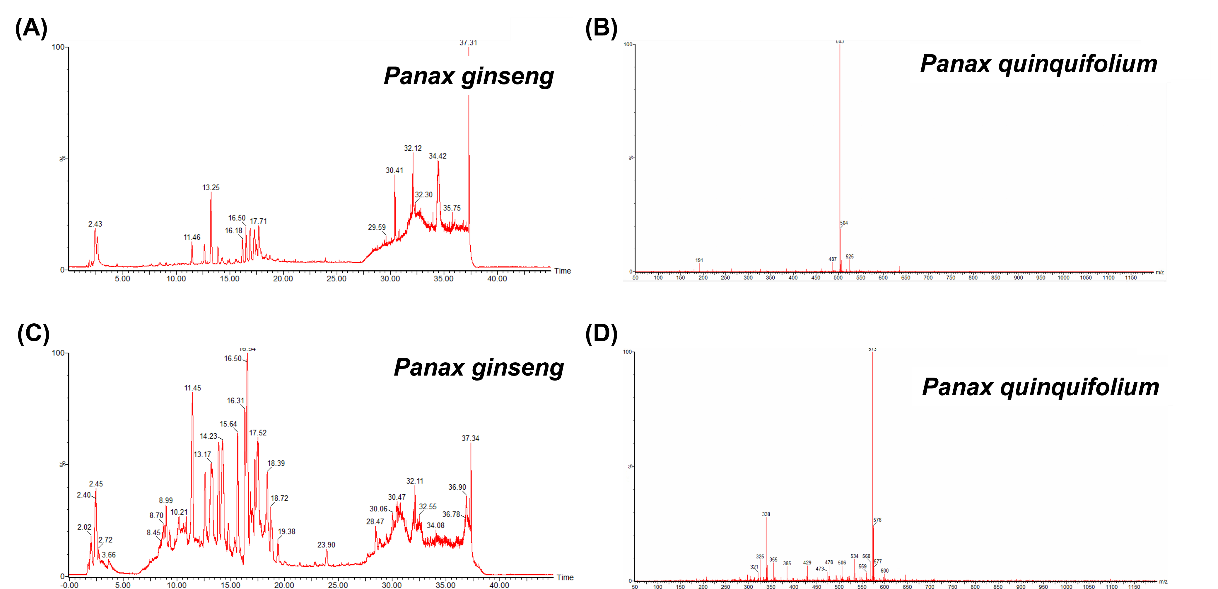
**

**Supplementary Figure 3**. Chromatograms of Korean and American ginseng. (A-B) MS/MS negative mode; (C-D) MS/MS positive mode

.
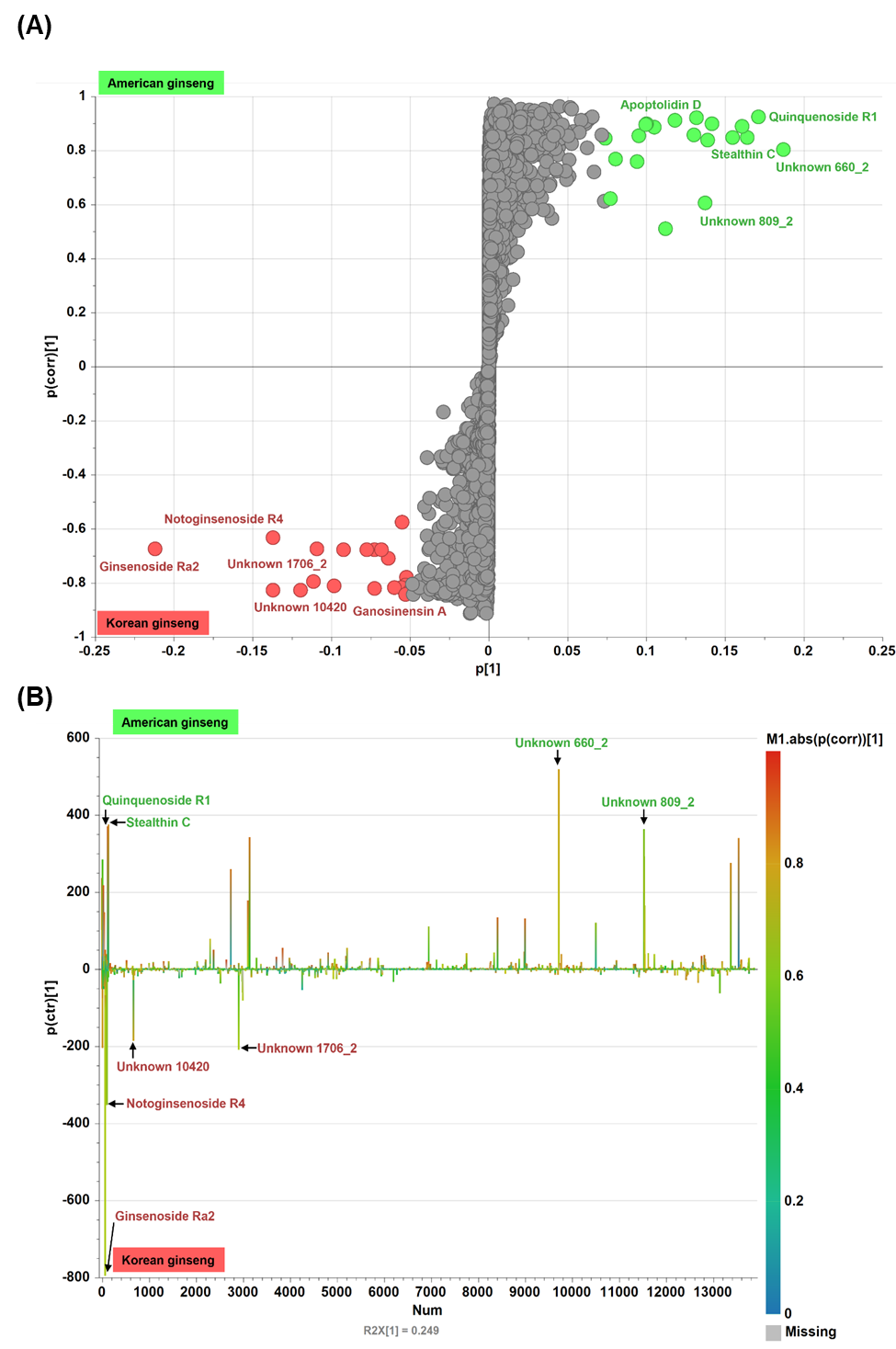


**Supplementary Figure 4.** (A) S-plot identifying discriminant metabolites including ginsenoside Ra2, notoginsenoside R4 (Korean ginseng), and quinquenoside R1 (American ginseng). (B) S-line plot highlighting characteristic ion features enriched in each species.


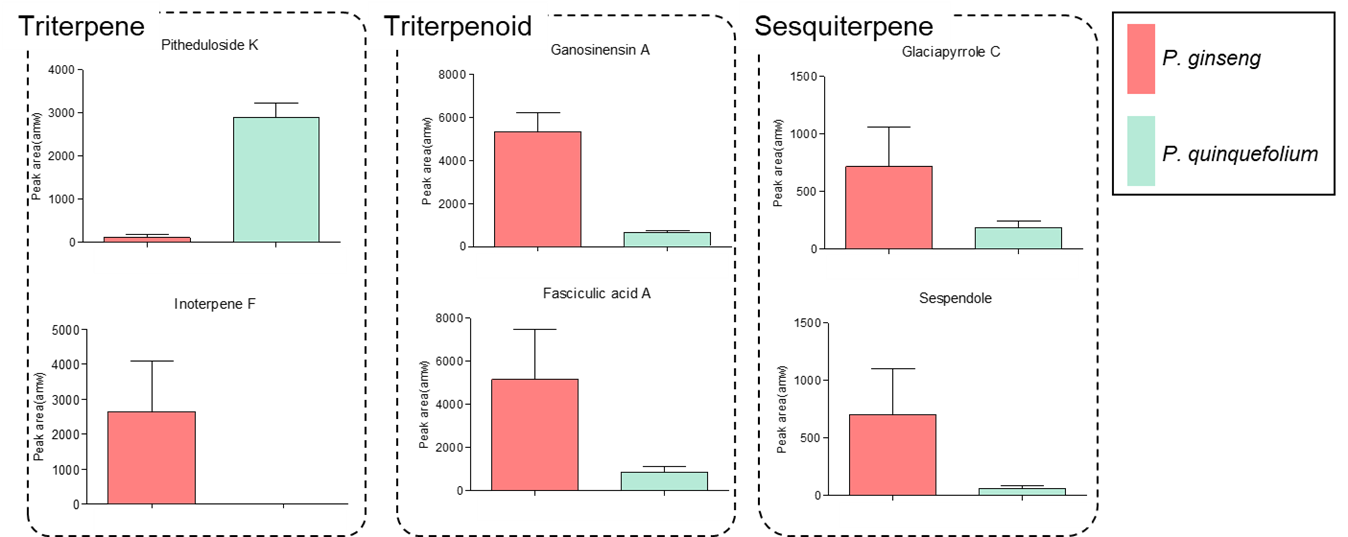


**Supplementary Figure 5.** Comparison of terpenoid-related metabolites in Korean and American ginseng.

**References**

Agius C, Von Tucher S, Poppenberger B and Rozhon W. Quantification of sugars and organic acids in tomato fruits. MethodsX. 5: 537–550 (2018).

Cajka T and Fiehn O. Toward Merging Untargeted and Targeted Methods in Mass Spectrometry-Based Metabolomics and Lipidomics. Analytical Chemistry. 88: 524–545 (2016).

Sławińska A, Jabłońska-Ryś E and Stachniuk A. High-Performance Liquid Chromatography Determination of Free Sugars and Mannitol in Mushrooms Using Corona Charged Aerosol Detection. Food Analytical Methods. 14: 209–216 (2021).

Wang L-H, Wen Y-J, Wang W-X, Zhai M, Li S-F, Zheng Q-X, Liu P-P, Zhang Y, Lv Y, Zhou H-N and Yu Y-J. A combination of automatic untargeted metabolic profiling analysis with targeted metabolomics to enhance geographical discrimination of Goji berry. Food Chemistry: X. 33: 103549 (2026).

Woyciechowski L, More TH, Kaltenhäuser S, Meller S, Zacharias K, Twele F, Dopfer-Jablonka A, Welte T, Illig T, Behrens GMN, Volk HA and Hiller K. Headspace SPME GC–MS Analysis of Urinary Volatile Organic Compounds (VOCs) for Classification Under Sample-Limited Conditions. Metabolites. 16: 57 (2026).
